# Disrupting the clock: meta-analysis of irregular night shifts and migraine, proposing shift work migraine disorder with chronobiology strategies

**DOI:** 10.3389/fneur.2025.1684169

**Published:** 2025-12-09

**Authors:** Yohannes W. Woldeamanuel, Ariana Rahman, Esam T. Hyimanot, Richa Chirravuri, Mahya Fani, Elika D. Javaheri, Madeline Welch, Joyce Zhuang, Chung Jung Mun

**Affiliations:** 1 Mayo Clinic Arizona, Scottsdale, AZ, United States; 2 Arizona State University, Tempe, AZ, United States; 3 Southwest Minnesota State University, Marshall, MI, United States; 4 University of California, Riverside, Riverside, CA, United States; 5 Department of Psychiatry and Behavioral Sciences, Johns Hopkins School of Medicine, Baltimore, MA, United States

**Keywords:** migraine, circadian, shiftwork, meta-analysis, systematic review, headache, chronobiology

## Abstract

**Background:**

Migraine is linked to circadian rhythm disruptions, with morning attack peaks, circadian variations in trigeminal pain sensitivity, anterior hypothalamus involvement, and core circadian clock gene activity. Irregular night shift work, affecting up to 50% of the population, including new parents and students, causes significant circadian disruption. We hypothesize that irregular night shifts increase migraine prevalence compared to fixed schedules.

**Methods:**

A systematic review and meta-analysis of observational studies up to March 27, 2025, assessed migraine prevalence in irregular versus fixed night shift workers, searching Web of Science and PubMed with terms like “shift work” and “migraine” (PRISMA/MOOSE-compliant, PROSPERO: CRD420250654865). Study quality was evaluated using the Newcastle–Ottawa Scale (NOS). A random-effects meta-analysis calculated weighted odds ratios (ORs) for migraine prevalence.

**Results:**

From 203 records, 13 high-quality cross-sectional studies (*N* = 38,798,271, 77% female, NOS 9–10) showed irregular night shifts significantly increased migraine odds (OR = 1.61, 95% CI: 1.27–2.04, *p* < 0.0001, *I*^2^ = 73%), with females at higher odds (OR = 2.02–4.21). Meta-regression linked higher female representation to increased migraine odds (*β* = 0.70, *p* = 0.0003, *R*^2^ = 50%). Irregular night shifts showed no association with tension-type headache (OR = 0.79, 95% CI: 0.43–1.45).

**Conclusion:**

Irregular night shifts disrupt circadian rhythms, elevating migraine odds but not tension-type headache, suggesting fixed schedules may reduce the burden. Chronobiology-informed management, including slow-rotating schedules (≥5 days with rest days), delay-directed rotations, timed light exposure, and ambient temperature regulation, needs testing to prevent ‘Shift Work Migraine Disorder,’ a proposed distinct migraine subgroup.

**Systematic review registration:**

PROSPERO, CRD420250654865.

## Introduction

Migraine is a complex and debilitating neurological disorder characterized by recurrent episodes of severe headaches, often accompanied by sensitivity to light, sound, and nausea (1). The pathophysiology of migraine is multifaceted, involving the interplay of various neural systems, including the trigeminal nerve, the brainstem, and the cerebral cortex (2). Recent studies have highlighted the critical role of circadian rhythms in migraine pathophysiology, suggesting a significant link between disruptions in internal biological processes and migraine attacks (3–9).

The observed circadian rhythmicity in migraine attacks exhibits distinct patterns, including matutinal/morning peaks in attack frequency, as well as circaseptan (weekly) and infradian (longer than 24 h) patterns (4, 7). Furthermore, circadian variations in trigeminal pain sensitivity have been observed, suggesting a complex interplay between circadian rhythms and migraine pathophysiology (10). The involvement of the anterior hypothalamus (11), home to the suprachiasmatic nucleus (SCN), the “master clock” regulating circadian rhythms, and the expression of circadian-related genes such as CK1δ, PER2, and RORα, provide additional evidence supporting the link between circadian rhythms and migraine (4, 12, 13).

The circadian system is a highly conserved and hardwired biological system that regulates various physiological processes, including pain sensitivity (14, 15), sleep–wake cycles (16), neurotransmitter and hormone secretion (17, 18), and metabolism (17, 19). Disruptions to this intricate system, whether due to lifestyle factors, environmental influences, or genetic predispositions, can have far-reaching detrimental consequences, contributing to the development of various chronic diseases, such as cancer (20–24), diabetes (19, 25–27), chronic pain (14, 28), cardiovascular disease (29), and neurological disorders such as Alzheimer’s disease (30–32).

Shift work involves organizing 24-h operations into two or three distinct shifts, with start and end times varying based on shift length (33). According to the US National Institute for Occupational Safety and Health (NIOSH), day shift typically runs from 5–8 a.m. to 2–6 p.m., evening shift from 2–6 p.m. to 10 p.m.–2 a.m., and night shift (colloquially known as “graveyard shift”) from 10 p.m.–2 a.m. to 5–8 a.m. (33). The International Labor Organization (ILO) (34) and International Agency for Research on Cancer (IARC) (35) define shift work as any work schedule outside conventional daytime hours, typically spanning 7:00 a.m. or 8:00 a.m. to 5:00 p.m. or 6:00 p.m., such as evening or night shifts. The ILO defines night work as any work performed during a period of at least seven consecutive hours, including midnight to 5 a.m., and a night worker as someone whose job involves a substantial amount of such hours exceeding a specified threshold (34). The European Union (EU) Working Time Directive (WTD) adopts the same definition of “night time” as the ILO and defines a “night worker” as an individual who regularly works at least 3 h of their daily shift during this period (36). Globally, approximately 20% of the workforce engages in shift work, with regional variations, such as 12% in Europe (up to 58% in evening work) (37) and 15% in Chile (38), while in the U.S., shift work prevalence is highest in service industries like protective services (50.4%) and food preparation (49.4%) (38). In other regions, night work affects 7.6% of workers in Brazil (39), 16% in Australia (40), 17.5% in China (41), 20% in Senegal (42, 43), 21.8% in Japan (44), and 28% in Canada (45), with significant prevalence in sectors like healthcare, hospitality, manufacturing, and transportation. Approximately 27% of the U.S. workforce reports engaging in evening or night shift work, with 7.4% specifically reporting frequent night shift work, defined as working between 1:00 a.m. and 5:00 a.m. for 6 to 30 days within the preceding 30-day period (46).

Migraine shows significant disparities in prevalence and severity across age groups (47, 48), sexes (48–50), and races (51–56). The specific impact of night shift work as a contributing factor to these disparities remains underexplored, warranting focused investigation.

- Age and night work prevalence: According to the NIOSH, the prevalence of frequent night work demonstrates a clear age-related pattern, with the highest rates observed in the youngest demographic: 18–29 years (8.61%). This prevalence gradually decreases with increasing age: 30–44 years (7.78%), 45–64 years (6.93%), and is lowest in individuals aged 65 and older (3.68%) (46). Similarly, as per the US Department of Labor (DOL) report, young workers (aged 15–24) are more likely to work non-daytime schedules, including evening, night, rotating, or irregular shifts, with 31.9% on such shifts compared to 16.4% of the total workforce (57).
- Sex differences: Females (5.57%) engaged in less night shift work compared to males (9.11%) (46). Studies show that females experience a migraine burden three times greater than males (48–50). This increased susceptibility in females is further exacerbated by a higher prevalence of psychological comorbidities, such as anxiety and depression, and shorter free-running circadian cycles (58). These factors may contribute to increased circadian disruption when subjected to night shift schedules, potentially amplifying the adverse effects on migraine.
- Racial disparities: Racial disparities in night work prevalence are important. In the NIOSH report, Black populations demonstrate the highest rates of night shift work (10.5%), followed by White populations (7.07%) and other racial groups (6.48%) (46). The DOL report shows that Black workers are more likely to work non-daytime schedules, with 24.1% on such shifts compared to 15.2% of White workers and 16.4% of the total workforce (57). Notably, research indicates that Black individuals tend to have shorter free-running circadian cycles compared to White individuals (58–60). This physiological difference may further exacerbate the detrimental effects of circadian disruption associated with night shift work, potentially intensifying health disparities. Black populations also have limited healthcare access, lower treatment rates, and greater functional disability due to more severe migraine pain, intensifying migraine burden and disparities (51–53, 56, 61, 62).
- Intersection of demographics, night shift work, and migraine: The demographic profiles of younger females and Black workers align with populations already identified as having a higher prevalence and burden of migraine. Previous studies have consistently documented elevated migraine burden, increased severity, and pronounced disparities within these groups (48, 50, 53, 55, 61). We hypothesize that frequent night work, with its inherent disruption of circadian rhythms, to be a significant trigger for both migraine exacerbation and *de novo* onset. Beyond ‘traditional’ shift work, students facing academic pressure and new parents experiencing fragmented sleep due to newborn care are particularly vulnerable to night shifting and its associated circadian rhythm disruption (63, 64). Further in-depth investigation into the impact of night work on migraine prevalence and severity across all affected populations is warranted to develop targeted interventions and mitigation strategies that benefit diverse demographic groups.

Rotating or irregular night shift work, characterized by unpredictable changes in shift timing, is linked to greater circadian disruption, long sleep, depression, anxiety, and fatigue compared to fixed night shift work, likely due to increased recovery needs (65–71). Rotating night shifts also significantly elevate psychological distress and impair sleep quality, posing substantial health risks (65, 67). Given the established link between circadian disruptions and migraine, we hypothesize that irregular or rotating night shift work, which is associated with increased circadian disruption, psychological distress, and reduced sleep quality compared to fixed night shift work, may correlate with a higher prevalence of migraine. Our objective is to investigate this relationship to inform evidence-based interventions aimed at reducing migraine burden in this vulnerable population.

## Methods

### Research question

Our research question followed the PECO (Population, Exposure, Comparison, Outcome) (19) format: general working population (P); irregular night shift work or rotating night shifts with ≥ 5 nights/month (E), defined by not remaining on the same schedule for ≥2 weeks; regular non-rotating night/evening or permanent night shifts with <5 nights/month (C); and migraine/headache prevalence (O), assessed via odds ratios from observational studies.

### Eligibility criteria

Studies were eligible if they included adult workers (≥18 years) from general populations (any occupation/industry; excluding clinical/non-working groups like patients/retirees). Exposure required frequent irregular/rotating night/evening shifts with schedule changes (34, 36); comparisons needed fixed non-rotating night/evening schedules. Evening/night shifts were grouped due to temporal overlap (e.g., 9:00 p.m.–midnight) and shared health effects.

While there’s overlap in the hours [9:00 p.m. to midnight are included in both evening and night shifts (33, 46, 57)], both evening and night shifts are categorized together in these studies due to shared characteristics and health implications. Both shift types occur during peak melatonin production and sleep propensity, contributing to similar health risks (72). Including both captures the broader impact of night shift work schedules on circadian disruption, despite overlapping hours. Outcomes of interest were the prevalence of migraine or severe headache, defined by clinical criteria (e.g., International Classification of Headache Disorders/ICHD-based questionnaire for migraine), medical interview, or self-report. We included studies reporting migraine prevalence or associations with shift work, even if they encompassed other headache subtypes [e.g., tension-type headache (TTH), chronic daily headache (CDH), or medication-overuse headache (MOH)]; we retained those with extractable migraine-specific data but also included severe headache or CDH-focused studies without explicit migraine subgroups as proxies for high-burden migraine-like presentations.

Studies were required to provide odds ratios (ORs) to quantify the association between shift work and migraine or headache prevalence, either directly reported ORs or data allowing for OR calculation (e.g., prevalence rates, contingency tables). Eligible study designs included observational studies, specifically cross-sectional studies, case–control studies, and cohort studies (prospective or retrospective), published in peer-reviewed journals or grey literature (e.g., theses, conference proceedings) with sufficient data, in any language (with translation available if needed), and with no date restrictions, given continuous knowledge updates. The 2015 NHIS report (46) was included in our systematic review as it provided relevant data on night shift work and migraine, aligning with our study’s focus.

Exclusion criteria were applied to maintain focus and quality. Studies were excluded if they exclusively involved non-workers (e.g., students, unemployed individuals, retirees), were limited to pediatric populations (<18 years) or specific clinical cohorts (e.g., only migraine patients without a working context), lacked clear definitions of night shift work (e.g., no mention of timing or irregularity), did not differentiate shift work (e.g., combining day and night shifts without separate analysis), or focused solely on day shift work without night shift comparison. Studies without a comparison group (e.g., case series or descriptive studies with no control) or those comparing night shift work to irrelevant groups (e.g., unemployed individuals) rather than the specified comparators were also excluded. Additionally, studies were excluded if they did not report migraine or headaches as outcomes (e.g., focusing only on sleep disorders or fatigue) reported only headache subtypes other than migraine (e.g., tension-type or cluster headaches), unless headaches were non-subtyped, or provided only qualitative outcomes (e.g., no prevalence or OR data). Studies not providing odds ratios or sufficient data to derive them (e.g., only *p*-values or narrative results), using experimental or non-associational metrics (e.g., means without prevalence), or employing non-observational designs (e.g., randomized controlled trials, laboratory-based experiments) were excluded. Reviews, editorials, or opinion pieces without original data were also excluded, though their reference lists were screened for eligible studies. Studies with insufficient detail on shift work patterns (e.g., no frequency or quick return data) were excluded. Including studies where ORs could be calculated from prevalence or contingency tables (not just reported) broadened the pool of eligible studies without sacrificing rigor. All reviewers used these criteria to screen titles and abstracts, then full texts, resolving discrepancies through consensus. The criteria ensured that studies aligned with the question’s focus on the impact of irregular night shifts on migraine and headaches, while excluding irrelevant or low-quality data.

### Search strategy and study selection

The process for identifying, screening, and selecting studies followed the Preferred Reporting Items for Systematic Reviews and Meta-Analyses (PRISMA) guidelines. The search strategy was tailored to the research question and the inclusion/exclusion criteria, specifying the databases, keywords, and time frame used to identify observational studies examining the association between irregular or rotating night shift work and the prevalence of migraine or non-subtyped headaches among workers, as measured by odds ratios or calculable odds ratios (e.g., from prevalence or contingency tables). The databases searched included PubMed and Web of Science. The search used a combination of controlled vocabulary (e.g., MeSH terms in PubMed) and free-text keywords to capture the population, exposure, comparison, outcome, and study design elements, grouped by concept and combined using Boolean operators (AND, OR, NOT). For the population (workers), terms included controlled terms like “Workers” [MeSH], “Occupational Groups” [MeSH], “Employment” [MeSH], and free-text terms such as worker*, employee*, “working population,” “labor force,” occupation*, staff, and personnel. For exposure (irregular or rotating night shift work), controlled terms included “Shift Work Schedule” [MeSH], “Work Schedule Tolerance” [MeSH], “Circadian Rhythm” [MeSH], and free-text terms like “night shift*,” “shift work,” “rotating shift*,” “irregular shift*,” “quick return*,” “short recovery,” “frequent shift*,” “shift change*,” and “night work.” Comparison terms (less frequent shifts, regular shifts, non-night work) included free-text terms such as “regular shift*,” “fixed shift*,” “non-rotating shift*,” “day shift*,” “permanent schedule*,” “non-night shift*,” and “standard work hours.” For the outcome (migraine and non-subtyped headaches), controlled terms were “Migraine Disorders” [MeSH], “Headache” [MeSH], “Headache Disorders” [MeSH], and free-text terms included migraine*, headache*, cephalalgia, “head pain,” and “cranial pain,” with a note that subtypes like “tension-type” or “cluster” would be excluded unless part of broader headache data. Measurement and study design terms (odds ratios, relative risks, observational studies) included controlled terms like “Odds Ratio” [MeSH], “Relative Risk” [MeSH], “Observational Study” [MeSH], “Cross-Sectional Studies” [MeSH], “Case–Control Studies” [MeSH], “Cohort Studies” [MeSH], and free-text terms such as “odds ratio*,” OR, prevalence, association, “cross-sectional,” “case–control,” cohort, observational, and epidemiology*.

**Figure 1:**
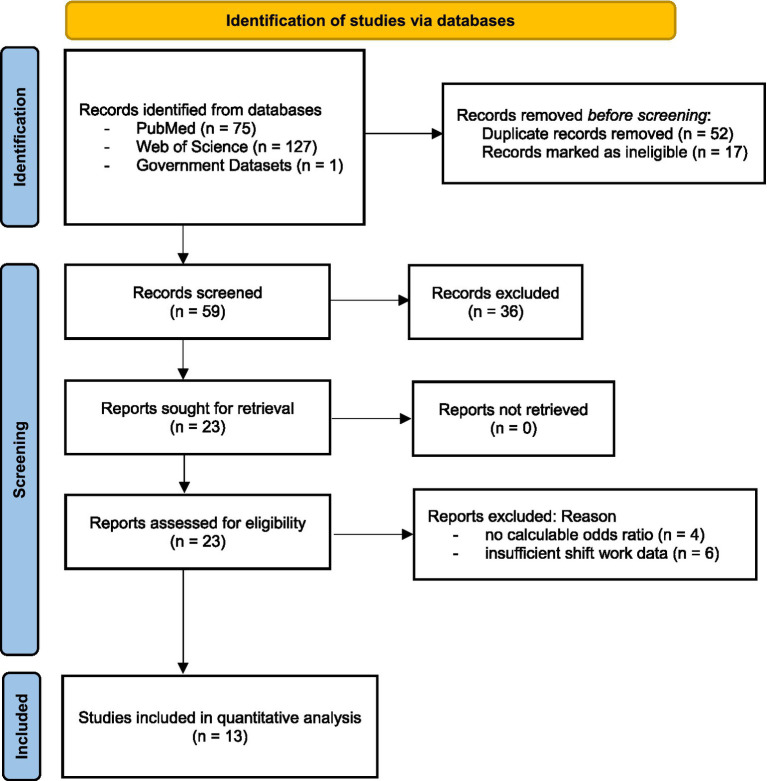
PRISMA flow diagram of study selection.

An example search string for PubMed was as follows: (“Workers” [MeSH Terms] OR “Occupational Groups” [MeSH Terms] OR “Employment” [MeSH Terms] OR worker* OR employee* OR “working population” OR occupation*) AND (“Shift Work Schedule” [MeSH Terms] OR “Work Schedule Tolerance” [MeSH Terms] OR “night shift*” OR “shift work” OR “rotating shift*” OR “irregular shift*” OR “quick return*” OR “night work”) AND (“Migraine Disorders” [MeSH Terms] OR “Headache” [MeSH Terms] OR migraine* OR headache* OR cephalalgia) AND (“Odds Ratio” [MeSH Terms] OR “relative risk*” OR “hazard ratio*” OR “Observational Study” [MeSH Terms] OR “Cross-Sectional Studies” [MeSH Terms] OR “Case–Control Studies” [MeSH Terms] OR “Cohort Studies” [MeSH Terms] OR “odds ratio*” OR prevalence OR “cross-sectional” OR cohort OR observational). A similar structure was adapted for Web of Science: TS = ((worker* OR employee* OR “working population” OR occupation*) AND (“night shift*” OR “shift work” OR “rotating shift*” OR “irregular shift*” OR “quick return*” OR “night work”) AND (migraine* OR headache* OR cephalalgia) AND (“odds ratio*” OR “relative risk*” OR “hazard ratio*” OR prevalence OR “cross-sectional” OR “case–control” OR cohort OR observational)). Filters in PubMed included study type (Observational Study, Cross-Sectional Studies, Case–Control Studies, Cohort Studies where applicable), language (no restriction, translations sought if needed), and species (humans), while Web of Science filters included document type (Article, Conference Proceeding, Early Access), research area (Medicine, Public Environmental Occupational Health, Neurosciences, Epidemiology if applicable), and language (no restriction). There was no date restriction, with searches including all available records up to the current date (March 27, 2025), reflecting the lack of a cutoff in the inclusion criteria and the continuous knowledge update capability, which ensures comprehensive coverage of both historical and recent studies. Search strings, run dates, and result counts were recorded for transparency (Figure 1, PRISMA flow diagram). Reference lists of included studies and relevant reviews were manually searched for additional eligible studies. This strategy strikes a balance between sensitivity (capturing relevant studies) and specificity (focusing on the question’s scope). We prioritized ORs for consistency across study designs, although cohort studies often report relative risks or hazard ratios. Therefore, we included terms for these metrics in our search and calculated ORs where possible.

### Data extraction

The following data were extracted: first author, year of publication, country, sample size, male to female ratio, study design, type of comparison (rotating vs. non-rotating night shiftwork, frequent vs. infrequent irregular night shift work), and number of people with migraine or headache (where headache was not phenotyped) in the compared groups. Six authors participated in data extraction.

### Quality assessment

The Newcastle–Ottawa Scale (73) (by YW and MF) was used to assess the quality of each included article in the following three domains: selection of study groups, comparability of groups, and ascertainment of exposure or outcome.

### Statistical analysis

A random-effects model was employed for the meta-analysis to account for between-study heterogeneity, which arises from differences in study settings, populations, and sample sizes. This model provides a more conservative estimate of the overall effect compared to a fixed-effects model. The inverse variance method with the DerSimonian-Laird estimator was used to weight studies based on their precision, accommodating the wide range of study sizes and heterogeneity effectively. In cases of sparse events, the robustness of this approach was considered, with the Mantel–Haenszel method as a potential alternative for sensitivity analyses to ensure stability in estimates. Odds ratios (ORs) were used as the effect measure instead of relative risk, as ORs provide consistent and comparable associations across observational studies, particularly cross-sectional designs. ORs are less sensitive to variations in baseline risks and are well-suited for studies where temporality or causality cannot be established. Data were synthesized using the online platform^1^ (74) to calculate effect sizes and assess heterogeneity. Meta-Essentials software (75) was utilized to perform statistical analyses, generate forest plots, conduct meta-regression, and evaluate publication bias. All procedures adhered to standard meta-analysis guidelines to ensure robust and reliable results. The study protocol was registered on PROSPERO (CRD420250654865) on 24 February 2025. Inter-rater reliability checks and duplicate screening were conducted by YW and EH to enhance methodological rigor.

## Results

*Study selection*: Figure 1 presents the PRISMA flow diagram detailing the study selection process. A total of (*n* = 203) records were identified from PubMed [(*n* = 75)] and Web of Science [(*n* = 127)], as well as government dataset (NHIS). After removing (*n* = 69) duplicates, 59 records were screened by title and abstract, excluding (*n* = 36) as irrelevant. Of 23 full-text articles assessed for eligibility, 10 were excluded for reasons including insufficient shift work data (*n* = 6) or no odds ratios or calculable data (*n* = 4). Ultimately, 13 studies (46, 76–87) were included in quantitative synthesis.

*Study characteristics*: The study analyzed a combined sample size of 38,798,271 participants, comprising 77% female participants. The participants originated from eight countries: China (3), Denmark (2), Saudi Arabia (2), Norway (1), United Kingdom (1), Singapore (1), Canada (1), United States (2). All included studies involved a cross-sectional design.

*Migraine and headache diagnosis*: Of the 13 included studies, eight employed migraine-specific diagnoses, while the remaining five used broader criteria for severe or chronic headache (Table 1). Five studies employed validated migraine diagnoses (e.g., ICHD-3 or physician interview/medical record review), while nine [including the NHIS 2015 (46)] relied on self-report measures.

**Table 1:** The risk of bias assessment shows Newcastle-Ottawa Scale (NOS) scores ranging from 9 to 10, indicating a high methodological quality.

		Selection				Comparability		Outcomes			
	Study	Q1	Q2	Q3	Q4	Q5a	Q5b	Q6	Q7	Total	Diagnosis
1	Affatato et al. (87)	1	1	1	2	1	1	2	1	10	Validated by physician diagnosis (migraine)
2	Al Maqwashi et al. (86)	1	1	1	2	1	1	2	1	10	Self-administered questionnaire (migraine)
3	Bjorvatn et al. (84)	1	1	1	2	1	1	2	1	10	ICHD-3b validated self-administered questionnaire (migraine, TTH, MOH)
4	Chan et al. (83)	1	1	1	2	1	1	2	1	10	Self-administered questionnaire (headache > once/week)
5	Jakobsen et al. (82)	1	1	1	2	1	1	2	1	10	Self-administered questionnaire (migraine)
6	Jensen et al. (81)	1	0	1	2	1	1	2	1	9	Self-administered questionnaire (headache)
7	Lees et al. (80)	1	0	1	2	1	1	2	1	9	Medical record review (headache)
8	Tasto et al. (78)	1	0	1	2	1	1	2	1	9	Self-administered questionnaire (CDH)
9	Wang et al. (77)	1	1	1	2	1	1	2	1	10	ICHD-3b validated self-administered questionnaire (migraine, TTH, CDH)
10	Xie et al. (76)	1	0	1	2	1	1	2	1	9	ICDH-3, neurologist interview validated, self-administered questionnaire (migraine, TTH)
11	Alturaiki et al. (85)	1	1	1	2	1	1	1	1	9	Self-administered questionnaire (migraine, TTH)
12	Liu et al. (79)	1	1	1	2	1	1	2	1	10	Self-administered questionnaire (headache)
13	National Institute for Occupational Safety and Health (46)	1	1	1	2	1	0	2	1	9	Interview (migraine or severe headache)
14	Molarius et al. (88)	1	1	1	1	1	1	1	1	8	Self-administered questionnaire (migraine/recurrent headache)

Most studies (7 of 13) scored a perfect 10 in selection, comparability, and outcomes (Q1–Q7). The other six studies scored 9, with minor deductions mainly in Q2 or Q6, reflecting slight variations in representativeness or exposure/outcome ascertainment. Molarius et al. (88) was included solely for direct OR comparison of female vs. male night-shift migraine risk (*n* = 10,503; 45% female), as it is one of only two studies providing disaggregated data. Of the 13 included studies, eight employed migraine-specific diagnoses, while the remaining five used broader criteria for severe or chronic headache. Five studies employed validated migraine diagnoses (e.g., ICHD-3 or physician interview/medical record review), while nine [including the National Institute for Occupational Safety and Health (46)] relied on self-report measures. Q stands for Question Number.

*Quality assessment* (Table 1): The Newcastle–Ottawa Scale (NOS) (20) scores for the listed studies range from 9 to 10, indicating high methodological quality across the board. The majority of studies (7 out of 13) scored a perfect score of 10, excelling in selection, comparability, and outcome criteria (Q1-Q7). The remaining six studies scored 9, with minor deductions primarily in Q2 or Q6, suggesting slight variations in representativeness or ascertainment of exposure and outcome.

**Figure 2:**
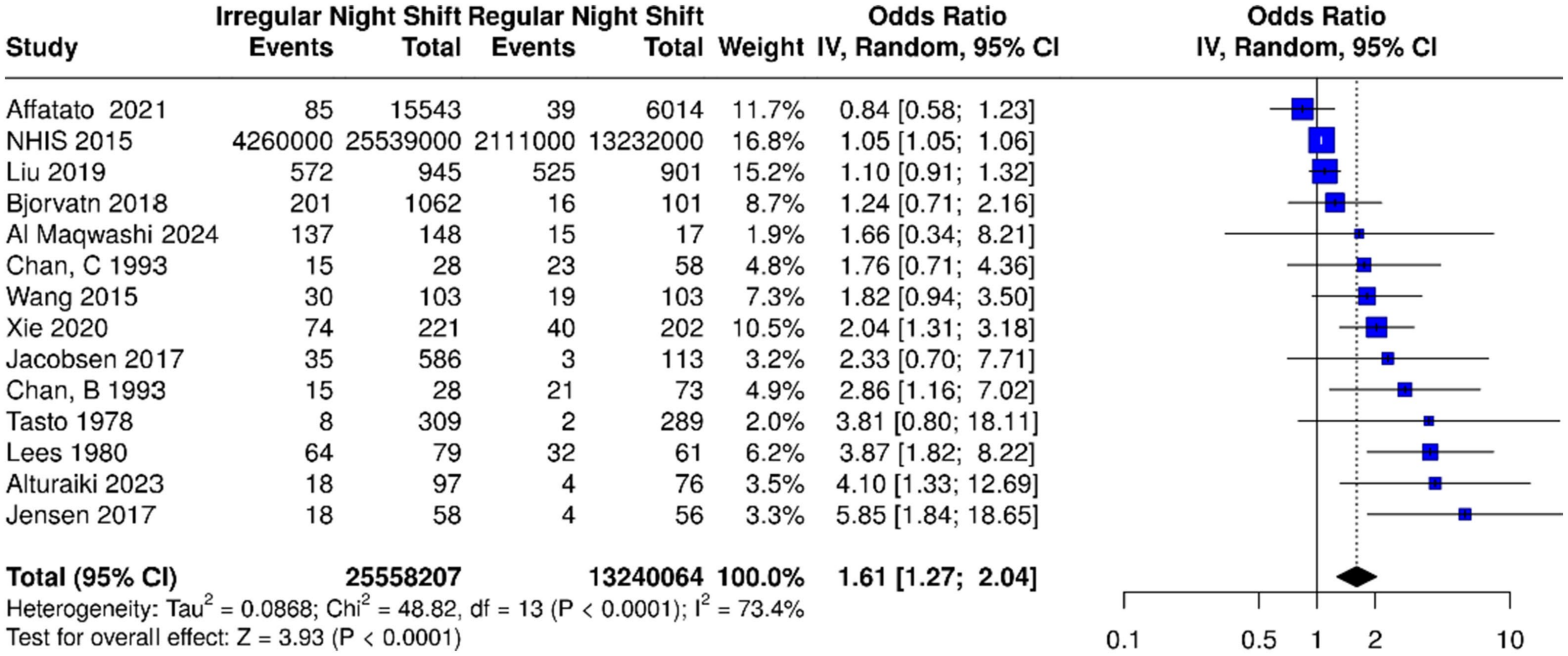
Based on the analysis performed using a random effects model with the inverse variance method to compare the odds ratio (OR), there was a statistically significant association between irregular night shift work and migraine prevalence; the summarized OR was 1.61 with a 95% confidence interval (CI) of 1.27–2.04 among the 13 studies [14 datasets including factories B and C from Chan et al. (83)]. Individual study estimates are depicted as squares (size proportional to weight), with horizontal lines for 95% CIs; the diamond summarizes the overall pooled OR and CI. IV, inverse variance; dF, degrees of freedom. Events represent the number of cases of migraine.

*Quantitative synthesis*: Altogether, 13 studies were analyzed. Based on the analysis performed using a random effects model with the inverse variance method to compare the OR, a statistical difference was observed; the summarized OR was 1.61 with a 95% confidence interval/CI of 1.27–2.04 (see Figure 2). The test for overall effect showed significance at *p* < 0.0001. The *I*^2^ value indicated a 73% inter-study heterogeneity.

*Sensitivity analysis*: Sensitivity analysis by diagnosis type yielded pooled ORs of 1.64 (95% CI: 1.02–2.69) for the five validated studies and 1.54 (95% CI: 1.16–2.05) for the eight self-report studies, with no significant subgroup difference (*p* = 0.83). Sensitivity analysis by specificity of diagnosis yielded pooled ORs of 1.40 (95% CI: 1.04–1.89) for the eight migraine-specific studies and 2.53 (95% CI: 1.30–4.93) for the five severe/recurrent daily headache studies, with a significant subgroup difference (*p* = 0.002). Excluding the NHIS study (which had a large sample size) resulted in a pooled OR of 1.88 (95% CI: 1.37–2.60) from the other 12 studies. Including it did not significantly change the results (*p* = 0.45).

**Figure 3:**
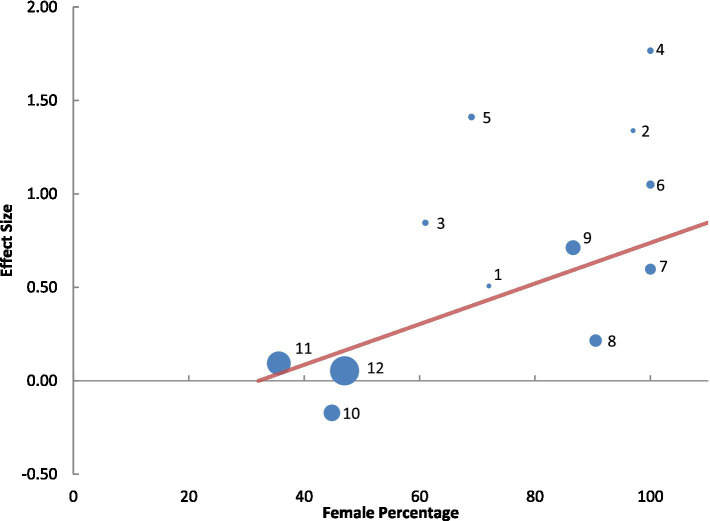
Meta-regression of 12 studies reporting male-to-female percentages, with bubble sizes indicating study weight and numbers corresponding to studies, showing a significant association between higher female proportion and increased migraine odds in irregular versus regular night shift workers (standardized *β* = 0.70, *p* = 0.0003), explaining 50% of the variance. Studies and their variance percentage contribution: 1, Al Maqwashi et al. (0.87%) (86); 2, Tasto et al. (0.92%) (78); 3, Jakobsen et al. (1.52%) (82); 4, Jensen et al. (1.62%) (81); 5, Alturaiki et al. (1.70%) (85); 6, Chan et al. (2.61%) (83); 7, Wang et al. (4.59%) (77), 8, Bjorvatn et al. (6.05%) (84); 9, Xie et al. (8.56%) (76); 10, Affatato et al. (10.68%) (87); 11, Liu et al. (21.94%) (79); 12, National Institute for Occupational Safety and Health (32.80%) (46).

Among the 13 studies reviewed, sex-specific data were available in only one study (87). An additional study, the only other in the literature providing disaggregated sex-specific data on night-shift migraine risk (88) was included outside the primary 13 studies, resulting in 10,503 participants (45% female) across these two studies. The OR for females was 2.02 (95% CI: 1.71–2.39) (88) in one study and 4.21 (95% CI: 2.09–8.46) (87) in the other, suggesting that females had approximately two to four times higher odds of migraine associated with night shift work compared to males. A meta-regression across 12 studies, which provided male-to-female percentage data, reinforced this finding. The meta-regression analysis revealed a significant association between the proportion of females and increased migraine odds in irregular versus regular night shift workers, with a *β* (standardized beta) of 0.70 (*p* = 0.0003), accounting for 50% of the variance (Figure 3).

**Figure 4:**
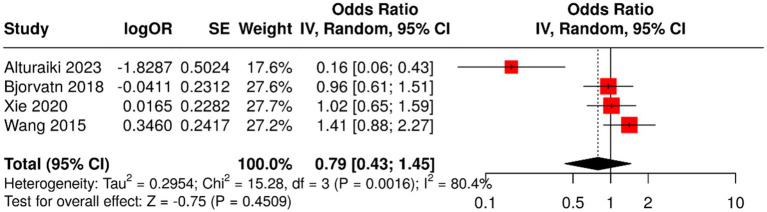
Meta-analysis of four studies using a random-effects model with inverse variance method to compare odds ratios (OR) of tension-type headache prevalence associated with irregular night shift work. The summarized OR was 0.79 (95% CI: 0.43–1.45), indicating no statistically significant association (test for overall effect, *p* > 0.05).

A meta-analysis of four studies with available tension-type headache (TTH) data, conducted using a random-effects model with the inverse variance method, evaluated the association between irregular night shift work and TTH prevalence. The pooled OR was 0.79 (95% CI: 0.43–1.45), indicating no statistically significant association (*p* > 0.05 for overall effect, *I*^2^ = 80%; Figure 4). Subgroup analysis showed a significant effect for migraine-only (subgroup 1) and all headaches (including non-phenotyped, subgroup 2). No significant differences were observed between migraine-only and all headaches subgroups (*p* = 0.25), with migraine appearing as the primary driver of the overall effect.

Meta-regression, using log-transformed (log10) sample size as a covariate to account for its wide range, showed no significant effect of sample size on the outcome (*β* = −0.31, *p* = 0.215).

*Sleep disturbances in included studies*: Among the eight studies reporting sleep data, significant disturbances were prevalent in shift workers, including insomnia [31.7% (84)], trouble initiating sleep [58% (81)], and overall sleep problems [~50% (79)]. These were associated with elevated migraine risk (OR: 1.43–2.64) and served as triggers [54.6% disturbances, 57.1% insufficient sleep (86)]. Short/long sleep durations further heightened odds [OR: 1.49–1.54 (82)]. No studies reported on sleep-affecting medications.

## Discussion

Night shift work, a necessity in many professions, disrupts the delicate balance of the circadian system. This disruption leads to physiological stress, irregular sleep patterns, and heightened sensitivity to migraine triggers. Our meta-analytic evidence suggests a robust association between irregular or rotational shift work and a greater prevalence of migraine. Our findings confirm that night shift work, particularly its irregular scheduling, is associated with an increased migraine burden. This discussion elaborates on the implications of fixed versus rotating shift schedules, female susceptibility to night shift effects, introduces the concept of “Shift Work Migraine Disorder” (SWMD) as a potential subgroup within the migraine spectrum, necessitating tailored interventions, and proposes strategies to reduce migraine exacerbation.

The comparable pooled ORs in self-report studies (1.54, 95% CI: 1.16–2.05) versus validated (1.64, 95% CI: 1.02–2.69; *p* = 0.83) affirm methodological consistency, indicating that self-report measures, while less rigorous, yield reliable estimates of shift work-migraine associations in population-based research. Self-report measures show a high level of agreement (~87%) with ICHD criteria in analogous population-based studies (89, 90). Sensitivity analysis excluding the NHIS study with the largest sample size yielded a pooled OR of 1.88 (95% CI: 1.37–2.60) from the remaining 12 studies, with no significant difference upon its inclusion (*p* = 0.45 for heterogeneity), underscoring the robustness of the overall estimate. The higher pooled OR in severe/recurrent daily headache studies (2.53, 95% CI: 1.30–4.93) versus migraine-specific ones (1.40, 95% CI: 1.04–1.89; *p* = 0.002) likely reflects inclusion of undiagnosed severe migraine cases—more vulnerable to circadian disruption—rather than TTH, which showed no shift work association, underscoring SWMD’s migraine-centric etiology and a dose–response gradient where broader criteria amplify observed effects.

### Fixed versus irregular shift schedules

Our results align with prior evidence that fixed night shift schedules are preferable to irregular or rotating schedules for minimizing circadian disruption (70). Irregular shifts, particularly those with rapid rotations [changing night shift schedules every 1–4 days (91)], quick returns [less than 11 h of rest between shifts (92)], and consecutive night shifts (>3 nights) (93, 94) desynchronize the SCN, the master circadian pacemaker, from peripheral clocks in organs such as the liver and gut (95). This desynchronization manifests as irregular sleep–wake cycles, and gastrointestinal disturbances, and may amplify sensory processing (3, 9, 96, 97) to migraine triggers such as bright light, loud sound, and dietary irregularities, though direct evidence is needed. Fixed schedules, by contrast, allow for gradual circadian adaptation for workers transitioning from day to night shifts, involving a 12-h schedule change, with the biological clock adjusting by approximately 1–2 h per day (60, 98). Based on this adaptation rate, we recommend maintaining consistent shift schedules for at least 12–14 days to allow sufficient time for entrainment of the 12-h shift, potentially reducing physiological stress and migraine risk, though further research is needed to confirm this effect.

Rotating shifts, when unavoidable, should follow a delay direction (morning → evening → night) rather than an advance direction (night → morning) (99–101). This aligns with the natural tendency of the human circadian clock to delay rather than advance, as demonstrated in a meta-analysis, which found lower rates of sleep disruption and mood disturbances with delay-rotated schedules (99–101). Additionally, minimizing consecutive night shifts—ideally to one per cycle—reduces cumulative sleep debt and cortisol dysregulation, both of which are implicated in migraine pathophysiology (102–106).

Avoiding quick returns (shifts with <11 h of rest) reduces fatigue and sleep disturbances, as short inter-shift intervals exacerbate circadian misalignment (92, 107). However, Katsifaraki et al. (108) found no association between sleep duration and headaches in shift workers (OR = 1.00, 95% CI: 0.97–1.02), suggesting headache triggers may involve circadian factors rather than sleep loss.

### Female susceptibility to night shift effects

Our meta-regression and subgroup meta-analysis of 10,503 individuals (45% female) reveal a significant sex disparity in night shift migraine risk, with females facing over twice the odds versus males. This aligns with women’s higher migraine prevalence, amplified by circadian disruption (50). Despite lower night shift participation (15.2% vs. 17.6% for males) (46), females bear greater shift work-migraine burden, underscoring the need to probe underlying mechanisms. This sex disparity may stem from inherent circadian differences, with females exhibiting a shorter free-running circadian period (24.2 vs. 24.5 h in males) (58, 109–111) that heightens vulnerability to night-shift desynchronization. Hormonal fluctuations (menstrual cycles, pregnancy, or menopause) exacerbate misalignment effects (112), while females show greater sensitivity to sleep deprivation and stress (113, 114), further compounded by caregiving duties (115, 116) and circadian-timed stress responses, as seen in female mice with exaggerated active-phase disruptions in clock genes (113, 114).

### Shift work migraine disorder: a proposed subgroup

Our study links irregular night shifts to higher migraine burden, proposing “shift work migraine disorder” (SWMD) as a novel chronobiological subtype warranting validation. We hypothesize chronic circadian misalignment amplifies trigeminovascular activation (117) via neuroinflammation and oxidative stress (118–120), evidenced by higher migraine prevalence in night vs. day workers and irregular vs. fixed night workers. SWMD likely increases susceptibility to circadian triggers, necessitating chronobiology-focused management. SWMD’s migraine specificity (vs TTH) aligns with greater sleep disturbances in migraine (121, 122); the absent TTH link underscores circadian disruption’s preferential migraine impact. Longitudinal studies should validate SWMD’s pathophysiology for potential ICHD inclusion, with proposed criteria:

A) Meets ICHD-3^1^ migraine criteria.
B) ≥3 months irregular night shifts [rotating shifts (91), quick returns (92), >3 consecutive night shifts (93, 94)].
C) Migraine onset/exacerbation temporally linked to shifts (during/within 24 h of changes).
D) Circadian evidence (actigraphy irregularities or daytime sleepiness).
E Not better explained by other disorders, including ruling out obstructive sleep apnea or periodic limb movements via polysomnography if clinically indicated.

These criteria distinguish SWMD from subtypes (e.g., chronic or menstrual migraine) by emphasizing circadian triggers, akin to shift work sleep disorder (SWSD) (123), a recognized circadian rhythm sleep disorder. Though sleep disruption triggers migraine (106, 124), SWMD uniquely ties attacks to shift transitions, differing from temporary jet lag disorder (125) and mirroring SWSD’s chronicity (Table 2). Unique factors include occupational context and melatonin suppression (72, 126–128), supporting targeted interventions and policies. Fixed schedules (e.g., 14-day consistency) and chronotherapy could aid alignment, unlike easier jet lag recovery, but require testing. In a study of 2,762 participants, social jet lag affected 18.9%, more in headache sufferers (21% vs. 17%, *p* = 0.006) (129), but not migraine-specific (22.4% vs. 20.8%, *p* = 0.651) (129) – indicating broad circadian effects without migraine distinction. Conversely, occupational SWMD merits separate recognition. Longitudinal validation and targeted interventions are essential; the Phase 3 solriamfetol trial for SWSD [NCT06568367 (130)] – a wake-promoting agent targeting TAAR-1, dopamine, and norepinephrine pathways overlapping with migraine mechanisms—may offer translational insights for SWMD due to shared circadian disruptions, but only as a hypothesis pending dedicated, migraine-specific efficacy trials.

**Table 2:** Summary of sleep disturbances in shift workers from included studies.

Study	Key sleep findings	Association with migraine/headache	Notes on medications/caffeine
Affatato et al. (87)	Sleep disorders were exclusion criteria.	N/A	No information on sleep medications.
Al Maqwashi et al. (86)	Sleep disturbances reported as a migraine trigger in 54.6% of participants; insufficient sleep in 57.1%.	High prevalence as triggers in migraineurs.	No information on sleep medications.
Alturaiki et al. (85)	No explicit sleep disorder data.	N/A	No information on sleep disorders or medications; caffeine use noted in 0.9% of migraine cases.
Bjorvatn et al. (84)	Insomnia prevalence: 31.7%.	OR = 1.55 (95% CI: 1.18–2.02) for migraine; OR = 1.01 (95% CI: 0.79–1.29) for tension-type headache.	No information on sleep medications.
Jakobsen et al. (82)	Short sleep (≤6 h: OR = 1.49, 95% CI: 1.21–1.85) and long sleep (≥9 h: OR = 1.54, 95% CI: 0.99–2.39) associated with higher migraine occurrence.	Associations persisted after adjustment for shift work.	No information on sleep medications.
Jensen et al. (81)	58% of nurses reported trouble falling asleep during night shifts.	Linked to shift work challenges.	No information on sleep medications.
Liu et al. (79)	~50% reported sleep problems; positive association with night shifts (OR = 1.43, 95% CI: 1.21–1.69).	OR = 2.64 (95% CI: 2.27–3.07) for headache.	No information on sleep medications.
Chan et al. (83)	Night shift workers had shorter sleep duration; rotating shifts had higher rates of poor sleep quality vs. controls.	Elevated poor sleep in rotating shift group.	No information on sleep medications.

OR, odds ratio; CI, confidence interval; TTH, tension-type headache. Data is limited to 8/13 studies reporting sleep metrics; there are no reports on sleep-affecting medications.

Recent within-person data from the 1,001 Nights cohort (131) bolster our SWMD proposal as a distinct chronobiological subtype, showing 31% higher headache prevalence on night shifts (aPR/adjusted prevalence ratio 1.31, 95% CI 1.13–1.52) persisting after adjustments for sleep, psychosocial stressors, and demands—echoing our meta-analysis OR of 1.61 for irregular shifts and emphasizing circadian misalignment over sleep disruption. Diary-based sleep controls (naps/latency, quality) uphold the association (aPR 1.31 from 1.33), affirming non-sleep-mediated hypothalamic-trigeminal disruption. Second-night peaks (aPR 1.54 vs. days, 95% CI 1.28–1.84) pre-adaptation refine phenotyping for early irregular exacerbations, aligning with our irregular/fixed split, endorsing slow rotations (≥5 days), and urging validation. Its female-dominant sample (*n* = 522, 14% migraine) improves our female predominance meta-regression (*β* = 0.70, *p* = 0.0003) generalizability via within-person design, advancing chronotherapy and ICHD-3 candidacy for preventable SWMD.

### Proposed strategies: how to work night shift and mitigate migraine burden

Beyond shift scheduling, our findings support multifaceted interventions, including chronotherapy (e.g., timed light) and policies such as mandatory recovery days, alongside sleep hygiene education (cool/dark environment, stimulus control, and sleep restriction) – proven effective in a meta-analysis for shift workers (132). Further research is needed on circadian-migraine links to refine strategies (Table 3).

A. *Photic circadian entrainment*

**Table 3:** Comparison of shift work related conditions: Jet lag disorder, shift work sleep disorder (SWSD), social jet lag, and proposed shift work migraine disorder (SWMD).

Feature	Jet lag disorder	Shift work sleep disorder (SWSD)	Social jet lag	Shift work migraine disorder (SWMD)
Cause	Time zone travel (more severe on traveling eastward) (125)	Night or rotating shifts (71, 123)	Misalignment between social and biological clocks (e.g., work vs. non-work sleep schedules) (129, 159)	Irregular night shift schedules
Duration	Temporary (125)	Long-term (123)	Chronic or recurrent (159, 160)	Chronic or recurrent
Symptoms	Insomnia, sleepiness, fatigue (125)	Insomnia, sleepiness, reduced alertness (123)	Sleep disruption, fatigue, mood changes (159, 160)	New onset or worsening migraine, characterized by increased frequency, severity, and heightened sensitivity to triggers (e.g., light, noise), associated with night shift work
Prevalence	Common in travelers (125)	27% (up to 49%) (123, 161–163) prevalence among night and rotating shift workers	21% in headache sufferers, similar in migraine vs. non-migraine headache sufferers (22.4% vs. 20.8%, p = 0.651) (129)	6 to 12% in evening and night shift workers (84); elevated migraine odds in irregular night shift workers (OR = 1.61), not TTH (OR = 0.79)
Severity	Mild to moderate (125)	Moderate to severe (123)	Mild to moderate (129, 159, 160)	Moderate to severe
Adaptation	Circadian alignment restores with time zone adjustment (125)	Circadian alignment difficult, requiring consistent schedules or interventions (123)	Persists without alignment of social and biological schedules (159, 160)	Circadian alignment challenging, may improve with fixed schedules (e.g., 14-day consistency)
Proposed treatment strategies	Timed light exposure, sleep schedules, melatonin supplementation (125, 164)	Fixed shifts, chronotherapy (e.g., light therapy), clockwise/delayed rotation, cool sleep environments, sleep hygiene (123, 165)	Consistent sleep schedules, limited weekend sleep variability (159, 160)	Fixed schedules (e.g., 14-day consistency), chronotherapy (e.g., timed light exposure), recovery days, clockwise/delayed rotation, cooling sleep environment; requires further study

Dynamic lighting mimicking day-night cycles (high melanopic lux 250–300 during shifts, dim <50 lux post-shift) reduces fatigue/mood issues (133), potentially stabilizing rhythms to cut migraine frequency. Blue-blockers post-shift reduce melatonin suppression (128); early-shift bright melanopic light (460-480 nm) boosts alertness (128, 134). Light therapy glasses (461 nm) cut sleepiness post-first shift (*p* = 0.012) (135), blue-enriched light (17,000 K) improves subjective alertness (136), and meta-analyses confirm benefits (134). Pre-Tmin bright light (7,000–12,000 lux, 2 h pre-wake) accelerate phase delays (133), while post-Tmin darkness (e.g., blue-blockers on commutes) aid adaptation and could reduce migraine – subject to verification (133, 137).

B. *Non-photic circadian entrainment*

Evening/nocturnal exercise (e.g., 19:00–22:00 or 00:30, moderate-high intensity) delays melatonin for better alertness/sleep (138–140). Time-restricted eating (10 h window post-shift) syncs metabolism, enhances cognition, cuts cardiometabolic risks (141–144). Cooler ambient temps (~23 °C) boost alertness, reduce discomfort, and adapt melatonin (145). Social interactions during/post-shift anchor rhythms (146–149), amplified by light strategies (150). Judicious caffeine (100-200 mg at onset) combats sleepiness (151) but risks daytime sleep disruption (152–154). Melatonin (0.5-3 mg, 1-2 h pre-sleep) aids re-entrainment (155, 156); a 2024 meta-analysis favors 4 mg 3 h pre-bed for optimization, with timing for phase shifts (morning delay, evening advance) (156, 157).

Sleep disturbances in 8 studies tie chronodisruption—through circadian misalignment—to migraine exacerbation, as irregular sleep rhythms heighten trigeminovascular activation, reinforcing our SWMD proposal. Incomplete sleep adjustments may still pose residual confounding in shift work-migraine associations, despite high NOS comparability (mean 9.6/10) indicating robust quality. The 1,001 Nights cohort (131) resolves this by confirming sleep non-mediation, with associations persisting (aPR 1.31) post-rigorous controls for sleep duration, quality, and timing. Chronic circadian misalignment from irregular night shifts in SWMD is hypothesized to disrupt the hypothalamic-trigeminovascular pathway, amplifying neurogenic inflammation and cortical spreading depression susceptibility, independent of sleep disruption—as evidenced by persistent associations post-sleep adjustment in cohort studies (131).

### Limitations

The moderate heterogeneity observed in our meta-analysis is scientifically reasonable, given the diversity of the included studies, i.e., different working populations, countries, settings, and time periods. A consistent direction of effect across most studies (as seen in the forest plot, with the majority to the right) supports the observed trend. Future trials should assess objective sleep assessments (158) to refine interventions. A key limitation is the heterogeneity in migraine diagnostic classifications across included studies—from rigorous ICHD-3 criteria or physician validation (*n* = 5) to unvalidated self-report (*n* = 9)—which may introduce misclassification bias by over-including non-migraine headaches or under-detecting subclinical cases, potentially yielding conservative pooled ORs. However, sensitivity analyses by validation type (OR 1.64 validated vs. 1.54 self-report, *p* = 0.83) and specificity (OR 1.40 migraine-specific vs. 2.53 severe headache, *p* = 0.002) confirm robustness, with the elevated severe headache estimate likely reflecting undiagnosed high-burden migraine cases more susceptible to circadian disruption, further supported by subgroup analyses showing significant shift work associations only for migraine (OR 1.40, 95% CI: 1.04–1.89) versus null for tension-type headache (OR 0.92, 95% CI: 0.71–1.19; *p* = 0.01 for difference), reinforcing the hypothalamic-trigeminovascular pathway’s selectivity.

## Conclusion

Night shift work poses a formidable challenge to the general population, potentially triggering migraine or exacerbating pre-existing ones. Evidence-based scheduling [clockwise rotations: morning–afternoon–night, favoring phase delays for better adaptation and longer rest (99, 100)] and circadian strategies, including sleep/light interventions, could optimize adaptation but require migraine-specific testing. With growing links between circadian health and chronic conditions like migraine, recognizing ‘shift work migraine disorder’ (SWMD) as a clinical entity is timely, warranting further study to enhance health for millions of night shift workers (99, 100).

## Data Availability

The original contributions presented in the study are included in the article/supplementary material, further inquiries can be directed to the corresponding author.

## Footnotes

1
https://metaanalysisonline.com/
